# Pediatric Rehabilitation of Acute Hyperextension-Induced Myelopathy After Surfing

**DOI:** 10.7759/cureus.48225

**Published:** 2023-11-03

**Authors:** Stephen E Ritter, Katee O'Malley, Kaycee Nguyen, Yousef Darwish, Nancy Yeh, Jace C Bradshaw, Adam Kenet

**Affiliations:** 1 Physical Medicine and Rehabilitation, Johns Hopkins University School of Medicine, Baltimore, USA; 2 Medicine, Texas A&M School of Medicine, Baylor University Medical Center, Dallas, USA; 3 Emergency Medicine and Anesthesiology and Critical Care, Johns Hopkins University School of Medicine, Baltimore, USA; 4 Biomedical Engineering, Johns Hopkins Whiting School of Engineering, Baltimore, USA

**Keywords:** acute hyperextension-induced myelopathy, myelopathy, surfer, ahim, pediatric rehabilitation, surfer's myelopathy

## Abstract

This case is a unique pediatric presentation of a surfer’s myelopathy, now referred to as acute hyperextension-induced myelopathy (AHIM), that provides an optimistic rehabilitation outcome. A 13-year-old boy presented to the emergency department with back pain, paraplegia, urinary retention, and dysesthesia following his first surfing lesson while visiting Hawaii. MRI of the thoracic spine without contrast showed a significant T2 hyperintense signal in the T9-T12 distal thoracic cord, consistent with AHIM. He completed a 10-day inpatient rehabilitation program and experienced exceptional improvement in functional mobility. AHIM is a rare phenomenon that is triggered by repetitive spinal hyperextension. While there are studies describing this clinical syndrome in detail, the literature lacks information about rehabilitation outcomes for these patients. Following the diagnosis and acute management of AHIM, a comprehensive inpatient rehabilitation program is recommended to maximize functional improvement.

## Introduction

This report presents the case of a young boy who was diagnosed with surfer’s myelopathy after his first surfing lesson. Surfer’s myelopathy, more recently described as acute hyperextension-induced myelopathy (AHIM), is a rare phenomenon triggered by repetitive spinal hyperextension [1-3]. The term “surfer’s myelopathy” was first described by Thompson et al. in 2004 by a case series of nine patients, ages 21-30, who developed a similar clinical syndrome with associated MRI findings of the lower thoracic cord after surfing in Hawaii [1]. Symptoms of the disorder include back pain followed by urinary retention, loss of sensation, and paraparesis or paraplegia [1-4]. The commonality between the patients included inexperience with surfing, no known predisposing history, and a lack of overt trauma [1,3]. The pathogenesis of this condition is believed to involve injury related to repetitive spinal hyperextension when quickly changing from lying prone to standing, paddling in a prone position, or hitting a wave [1,2]. These actions may cause compression of the central artery of Adamkiewicz or its radicular arteries, resulting in ischemia and infarction in the distal thoracic watershed zone of the spinal cord [1-3]. This condition is most commonly observed in inexperienced surfers but may also be seen in other sports, such as gymnastics, swimming, or golf [2]. Although there are studies describing this clinical syndrome in detail, the literature lacks information about the rehabilitation outcomes for these patients.

## Case presentation

**Figure 1:**
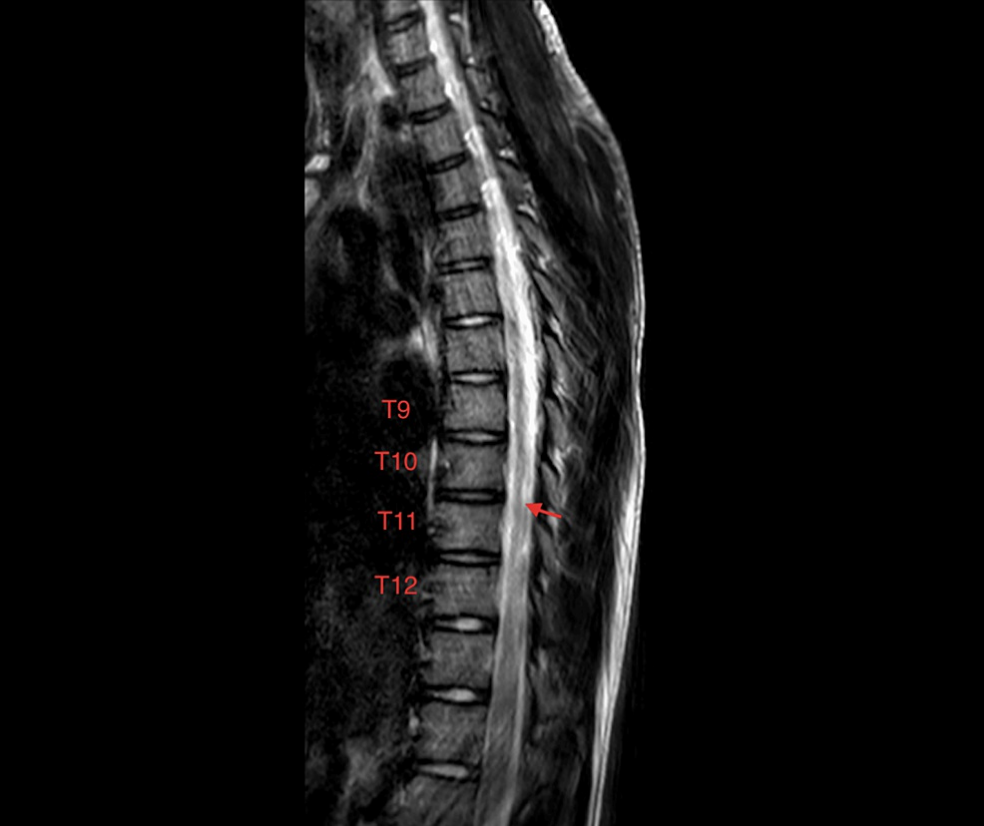
T2 weighted sagittal MRI images demonstrate hyperintense signal in the distal thoracic cord from T9-T12

**Figure 2:**
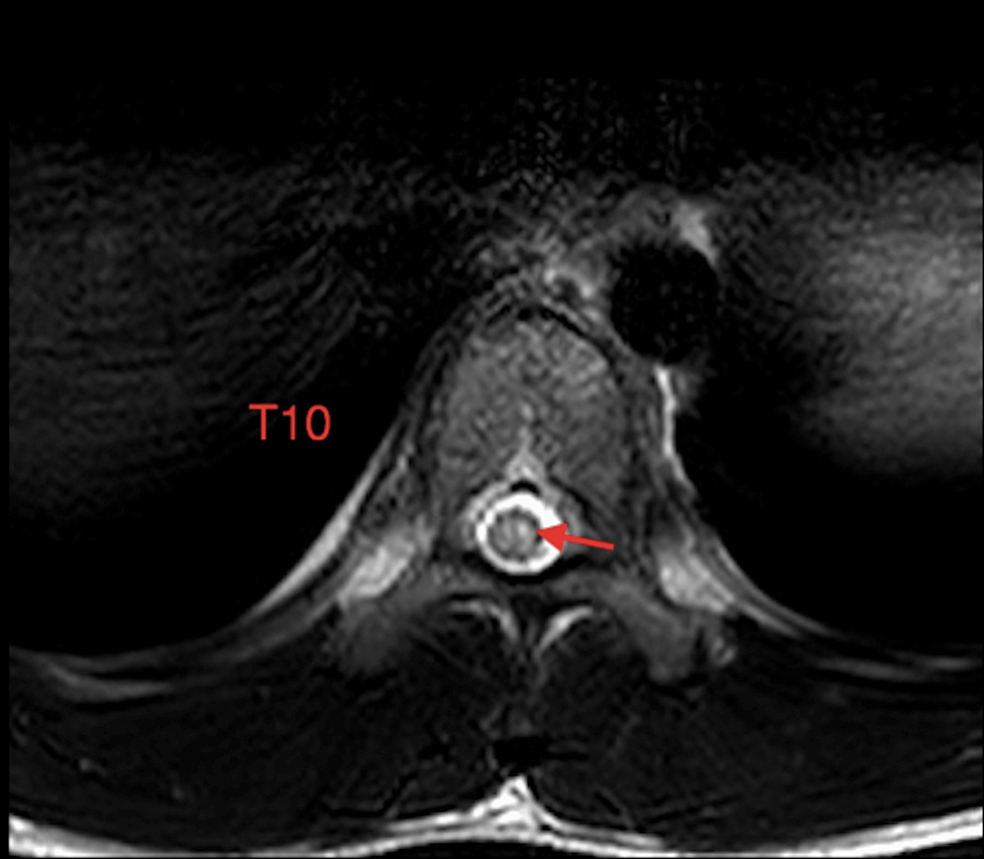
T2 weighted axial MRI image demonstrates high signal intensity in the central cord at the level of T10

The child described is a Chinese-American boy with a medical history of hypothyroidism. At baseline, he was athletic, played multiple sports, and had normal development (BMI: 19.5 kg/m2, height: 160 cm, weight: 49.9 kg). During his first surfing lesson, he developed lower back pain after approximately 30 minutes. One hour after completing the lesson, the child’s pain became severe, and he developed lower extremity weakness and dysesthesia. He did not recall any particular traumatic event during or preceding the lesson. In the emergency room two hours later, he exhibited substantial lower extremity paresis with only minimal bilateral hip flexion (manual muscle testing graded 1/5). He presented with severe lower back pain, described as “tingling” with radiation to both lower extremities, along with significant urinary retention requiring a Foley catheter. Patellar reflexes were absent, but Achilles reflexes were preserved. A Babinski test revealed bilateral upgoing toes. The sensation was preserved at the T10 level but was impaired in the bilateral lower extremities. MRI of the thoracic spine without contrast showed a significant T2 hyperintense signal in the T9-T12 distal thoracic cord (Figures 1-2). An MRI of the lumbar spine revealed no abnormalities.

The child was subsequently diagnosed with AHIM. The formal American Spinal Injury Association Impairment Scale (AIS) was not completed, but his documented physical exam upon presentation was most consistent with T10 AIS B. He was administered six milligrams of intravenous dexamethasone and transported to the pediatric intensive care unit. Although there is a lack of a clear guideline-directed protocol, the patient was treated with blood pressure augmentation. To maintain a mean arterial pressure goal of 70-85 mmHg, he received intravenous dopamine and phenylephrine for three days for induced hypertension. He showed clinical improvement over the subsequent days, including notable improvement in hip flexion to at least anti-gravity, the return of normal thigh sensation, and intact patellar reflexes. For pain management, he initially required morphine but eventually transitioned to acetaminophen only. Polyethylene glycol was also initiated due to concern for the neurogenic bowel. The child was stabilized, taught self-catheterization, and took commercial travel home for rehabilitation.

Upon admission to inpatient rehabilitation, the child exhibited poor eccentric control and ambulatory dysfunction. Using the Functional Independence Measures scale from complete independence to total assistance, the patient's function was subjectively graded by trained rehabilitation therapists throughout his stay. He initially required moderate assistance to perform transfers, including sit-to-stand, stand-to-sit, and stand pivot transfers. He could walk 56.7 feet with minimal assistance using a rolling walker for upper-extremity support and required maximal assistance for toileting. He completed a 10-day inpatient rehabilitation program and experienced exceptional improvement in functional mobility. Physical therapy focused primarily on lower extremity strengthening exercises, stretching, gait training, stair climbing, and aquatic therapy. Occupational therapy specifically focuses on standing balance, coordination exercises, household transfers, and improving independence for activities of daily living. Upon discharge, he had progressed to ambulating 1000 feet with contact guard assistance and supervision with a gait belt and had transferred independently. All activities of daily living were performed at a modified, independent level. Urinary retention improved with the use of a timed voiding schedule. Ten months after the initial injury, the patient had completed outpatient therapy for multiple sessions weekly and returned to participation in sports.

## Discussion

Data pertaining to long-term rehabilitation outcomes after AHIM are limited. In a 2022 retrospective systematic review of 104 patients with suspected AHIM, Alva-Diaz et al. proposed diagnostic criteria [2]. Differential diagnoses that may be considered include transverse myelitis, multiple sclerosis, spinal cord infarction, vasculitis, or coagulopathy [2]. The patient in this study met all five of the Alva-Diaz diagnostic criteria. MRI findings, although variable, tend to exhibit a typical T2 hyperintensity infarction pattern, such as a “pencil-shaped” hyperintense signal in the lower thoracic region or an “owl/snake eye” pattern of signal abnormality on axial views [2]. Treatment with intravenous corticosteroids or blood pressure augmentation may be used in the acute clinical setting, but the benefits of these treatments remain unclear [2,3]. Chang et al. found admission severity of spinal cord injury to be most predictive of neurological outcome [3]. While some patients may experience partial or full recovery, complete paraplegia can occur [1].

In 2013, Takakura et al. reported the disastrous consequences of three surfers who developed complete paraplegia from AHIM [5]. Despite limited reporting statistics regarding recovery, data from the retrospective review by Alva-Diaz et al. showed that more than half of the patients had partial or complete recovery, while 27 patients (26%) showed no improvement [2]. There was statistical significance between AIS A and no improvement (p=0.022), consistent with the findings of Chang et al. [2,3].

Aoki et al. (2013) studied the long-term rehabilitation course of a 26-year-old male diagnosed with AHIM classified as T12 AIS A given complete paraplegia [6]. Rehabilitation goals for this patient were to achieve modified independence with the use of a wheelchair [6]. Although the patient experienced moderate recovery of deep sensation in his lower extremities, he remained paraplegic. However, he was able to achieve modified independence with a wheelchair after four months. Despite a poor prognosis with a complete spinal cord injury classification, long-term rehabilitation was recommended [6].

## Conclusions

Given the rarity of AHIM and emerging research on this condition, there is limited data regarding rehabilitation outcomes. Though this study pertains to only one subject, this pediatric case of incomplete spinal cord injury provides an optimistic result and emphasizes the importance of early rehabilitation. Following the diagnosis and acute management of AHIM, a comprehensive inpatient rehabilitation program is recommended to maximize functional improvement. In the future, research studying larger cohorts is required for improved understanding regarding prognosis and rehabilitation outcomes in patients with this condition.
